# Noise Source and Individual Physiology Mediate Effectiveness of Bird Songs Adjusted to Anthropogenic Noise

**DOI:** 10.1038/s41598-018-22253-5

**Published:** 2018-03-02

**Authors:** Claire M. Curry, Paulson G. Des Brisay, Patricia Rosa, Nicola Koper

**Affiliations:** 1 Natural Resources Institute, University of Manitoba, 70 Dysart Road, 303 Sinnott Building, Winnipeg, Manitoba R3T 2M7 Canada; 2 Oklahoma Biological Survey, University of Oklahoma, Norman, OK USA

## Abstract

Anthropogenic noise is a pervasive pollutant altering behaviour of wildlife that communicates acoustically. Some species adjust vocalisations to compensate for noise. However, we know little about whether signal adjustments improve communication in noise, the extent to which effectiveness of adjustments varies with noise source, or how individual variation in physiology varies with response capacity. We played noise-adjusted and unadjusted songs to wild *Passerculus sandwichensis* (Savannah Sparrows) after measurements of adrenocortical responsiveness of individuals. Playbacks using songs adjusted to noisy environments were effective in restoring appropriate conspecific territorial aggression behaviours in some altered acoustic environments. Surprisingly, however, levels of adrenocortical responsiveness that reduced communication errors at some types of infrastructure were correlated with increased errors at others. Song adjustments that were effective in communicating for individuals with lower adrenocortical responsiveness at pumpjacks were not effective at screwpumps and vice versa. Our results demonstrate that vocal adjustments can sometimes allow birds to compensate for disruptions in communication caused by anthropogenic noise, but that physiological variation among receivers may alter effectiveness of these adjustments. Thus mitigation strategies to minimize anthropogenic noise must account for both acoustic and physiological impacts of infrastructure.

## Introduction

Anthropogenic noise from industrial activities such as petroleum extraction^1^ is widespread and alters soundscapes, behaviour and stress responses in many wildlife species^2–4^. This acoustic pollution could result in extensive impacts to wildlife in critically threatened ecosystems such as grasslands^5^. Noise can impact fitness by altering physiological costs^2^ and disrupting behaviours crucial for defending territories and attracting mates^6–8^ by preventing signals from being detected or recognised^9^. However, these effects may vary among industrial activities, as spectral characteristics of noise produced by different activities can vary greatly^4,10^. This suggests that ecological impacts of many different industrial activities might be mitigated by preferentially implementing infrastructure that produces noise at frequencies and amplitudes that produce the least disturbance and allow for the most compensatory behaviours from nearby animals.

Vocalisations can be altered to make signals audible in noisy environments^9,11^ but this can change signal content^6–8^ and compromise communication efficacy^12^. While many studies have demonstrated that the signalling animals can alter vocalisations to compensate for noise^10–13^, the literature has only recently focused on effects of anthropogenic noise on receivers^8,9,14–21^. Thus, less is known about whether signal adjustments actually improve communication, if efficacy varies with noise source and how this interacts with intrinsic individual variation to explain capacity for populations to adjust to noise. Thus, why animals show variable behavioural responses to different types of noise^2,10,22^ is not well understood. Extrinsic characteristics related to sound physics, particularly noise amplitude and frequency overlap between noise and acoustic signals, have been considered in some depth, but this does not always explain why signal alterations are necessary and effective in some systems^8^ but are ineffective^7,16^ in others.

Behavioural responses to acoustic signals are mediated not only by the signalling environment, but also by physiological mechanisms^23^, which can both affect behavioural response patterns to novel stimuli^22^ and be affected by exposure to chronic human disturbances^3^. Indeed, noise type can alter the glucocorticoid stress response^24^. Thus, differential physiological responses to infrastructure with different physical footprints^25^ and noise spectra might explain why some industrial noises have greater impacts than others. Acute changes in corticosterone levels (hereafter, CORT) can be used as an index of adrenocortical responsiveness. CORT is associated with territorial defence^26^, interacts with testosterone to regulate levels of territorial aggression during breeding^27,28^ and is known to increase in response to environmental perturbations^23,24^. Therefore, spectral characteristics of noise and altered adrenocortical responsiveness may both explain variation in responses to particular noise types and interact to explain why song alterations in the presence of noise improve communication at some types of infrastructure but not others.

**Figure 1:**
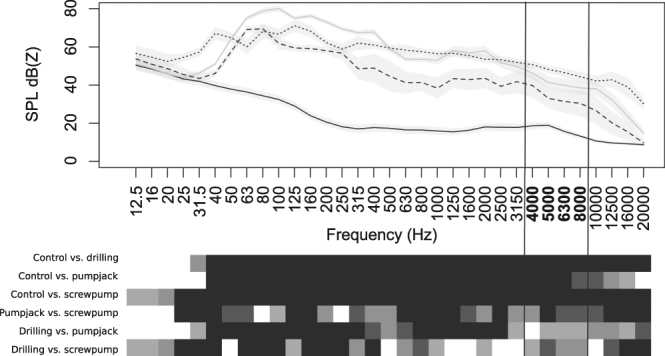
Frequency profiles for acoustic environments. Mean sound pressure levels (error bars 95% c.i.) at 10 m (Z-weighted time-average value in decibels, LZ_eq_) for each 1/3-octave frequency band 12.5–20,000 Hz. Symbols show background (i.e. control environments, black solid line; *n* = 50 at 46 sites), drilling playback (in which adjusted songs were recorded, grey solid line; *n* = 36 at 3 sites), pumpjacks (dashed line; *n* = 15 at 4 sites) and screw pumps (dotted line; *n* = 17 at 5 sites). Frequencies differ where cells below are black (*P* ≤ 0.0001), dark grey (*P* ≤ 0.001), medium grey (*P* ≤ 0.01) and light grey (*P* ≤ 0.05). Bold frequencies on the x-axis, bounded by vertical lines, indicate which frequencies are within the range of typical unadjusted Savannah Sparrow songs. Sound pressure levels were measured with a Brüel & Kjær 2250 SPL meter-frequency analyser (Brüel & Kjær, Denmark) along transects away from each site centre. More details on sound measurements are given in Curry *et al*.^57^ and Rosa *et al*.^56^.

We tested whether song adjustments to noise varied in effectiveness with infrastructure and physiology using an experimental design that combined playbacks of noise-adjusted and natural (unadjusted) songs in three environments, with responses of *Passerculus sandwichensis* (Savannah Sparrows) with naturally varying CORT levels. We colour-banded and sampled CORT using a 12-min standardised stress handling protocol^31^ from 35 free-living adult male Savannah Sparrows near Brooks, Alberta, Canada, in mixed-grass prairies. Birds held territories within control sites (11 males at 3 sites) and in noisy sites that contained two types of generator-powered oil wells with differing noise spectra: pumpjacks (12 males at 3 sites) and screw pumps (12 males at 3 sites). We predicted that these well types might have different impacts on stress and behaviour because pumpjacks are taller (4.5 m), move rhythmically along a vertical axis and produce noise with a different power spectra (Fig. 1) compared with screw pumps, which are also shorter (2.7 m) and have a horizontal spinning mechanism. Pumpjacks produce noise with significantly lower sound pressure levels than screw pumps, particularly in the frequency range that overlaps with Savannah Sparrow songs (Fig. 1). Savannah sparrows adjust their songs at both infrastructure types^29^. A mean of 14.0 ± 12.9s.d days (range 0–49.0) after colour-banding and CORT blood sampling, we played noise-adjusted and unadjusted songs to each colour-banded male and summed conspecific territorial aggression behaviours (hereafter “agonistic responses” for brevity) in six categories when the bird approached <20 m of the playback speaker: numbers of songs, calls, attacks (attacking speaker or flying over speaker) and wing flicks (an agitated movement); distance of closest approach; and time to closest approach.

Addressing how agonistic behaviour changes relative to reference baselines (playback of unadjusted songs in natural, quiet environments) adds to our growing knowledge of receiver perception of signals in altered landscape. Our use of CORT as a covariate helps further explain variation in behavioural responses, as CORT may be associated both with potential stress from infrastructure and with the behavioural responses themselves. Finally, associating CORT with behaviours in altered environments can suggest future areas of research into the mechanisms driving differential responses to adjustments made to varying acoustic environments.

## Results

At control sites, noise-adjusted songs resulted in different agonistic responses than unadjusted songs, suggesting that receivers interpreted content differently between unadjusted and adjusted songs (Table 1). Adjusted songs in control environments elicited more calls and fewer attacks and wing flicks.

**Table 1:** Responses to simulated territorial intrusions with noise-adjusted songs in control environments results in atypical responses for several behaviours.

	Number of songs	Number of calls	Number of attacks	Number of wing flicks	Min approach distance	Ln time to min approach
Song type (adjusted vs. unadjusted)	−1.16 ± 0.93 (0.214)	**8.27 **±** 3.45 (0.017)**	**−1.44 **±** 0.45 (0.001)**	**−0.94 **±** 0.42 (0.025)**	2.76 ± 2.8 (0.351)	−0.04 ± 0.2 (0.833)
CORT	−0.41 ± 1.22 (0.739)	**16.16 **±** 7.74 (0.037)**	0.66 ± 0.61 (0.277)	0.05 ± 0.73 (0.943)	0.69 ± 2.98 (0.819)	**−1.15 **±** 0.37 (0.009)**
Song type x CORT	**3.86 **±** 1.47 (0.008)**	**−15.58 **±** 7.81 (0.046)**	0.93 ± 0.61 (0.126)	1.24 ± 0.69 (0.071)	−3.79 ± 3.72 (0.335)	0.12 ± 0.26 (0.669)

Results are given as coefficient ±s.e. (*P*), with *P* ≤ 0.05 in bold. Categorical variables are shown with 1 vs. 0 (i.e. adjusted = 1 and unadjusted = 0), such that a positive β indicates an increase with category 1.We fit generalised linear mixed models with song played, adrenocortical responsiveness and two-way interactions as fixed effects, with male ID as a random effect, using only data from control sites. Playbacks (*n* = 19) to individuals (*n* = 11) were divided by song type: unadjusted songs at control sites (*n* = 10) and adjusted songs at control sites (*n* = 9).

**Figure 2:**
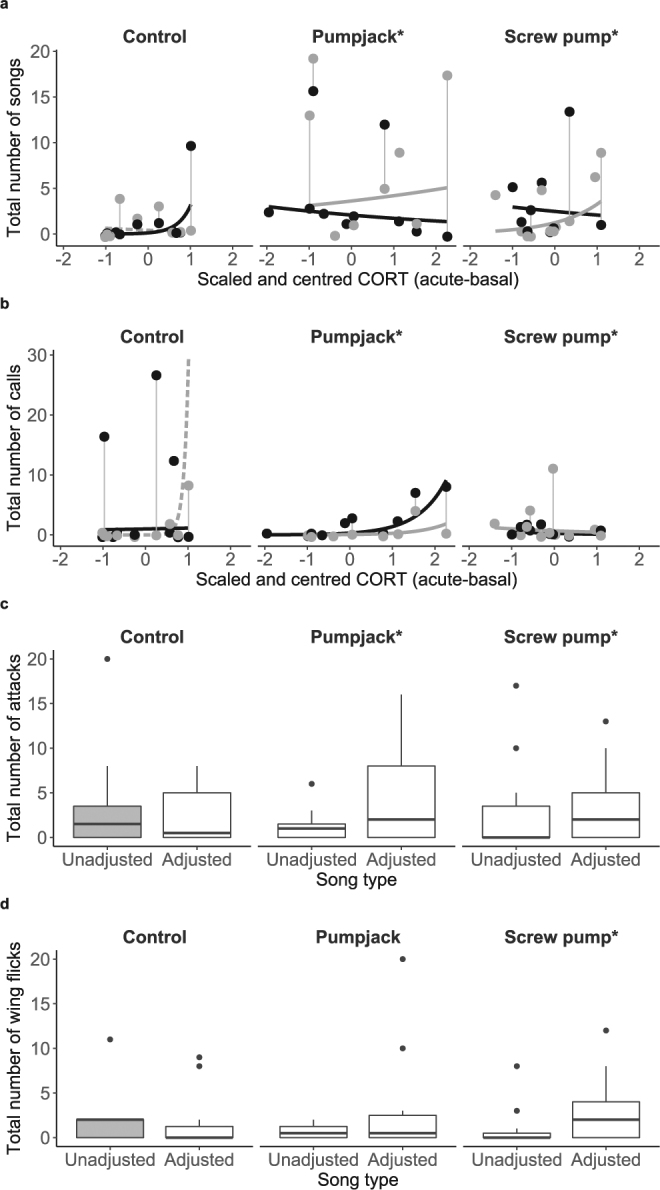
Responses to acoustically appropriate songs varied with treatment and adrenocortical responsiveness. Panels marked *show significant interactions from Table 1: three-way (**a**,**b**) or two-way (**c**,**d**) interactions relative to reference (unadjusted song at control sites). Reference (unadjusted songs in control sites) shown by grey dashed line and grey-filled box plots. (**a**,**b**), Vertical lines connect playbacks for each individual. Remaining lines (model-predicted values for treatments) and symbols (one playback) are responses to unadjusted (grey) or adjusted (black) songs. (**a**), songs. (**b**), calls. (**c**,**d**), box plots showing quartiles, median and outliers (dots) by song and infrastructure type for (**c**), attacks. (**d**), wing flicks.

In noisy environments, agonistic responses to unadjusted songs were inappropriate (different from unadjusted songs at control sites), but adjusted songs were effective in restoring several appropriate agonistic responses (Table 2; Fig. 2). The response to vocalisations in noisy environments was significantly more similar to the reference response when birds heard adjusted songs than when they heard unadjusted songs for three behaviours (Table 2): number of calls (Fig. 2b), number of attacks (Fig. 2c) and number of wing flicks (screw pumps only; Fig. 2d). Adjusted songs resulted in a more atypical response for minimum approach distance (pumpjacks only; see Supplementary Fig. S2).

**Table 2:** Responses to simulated territorial intrusions depended on song’s match to acoustic environment and adrenocortical responsiveness of individuals.

	Number of songs	Number of calls	Number of attacks	Number of wing flicks	Min approach distance	Ln time to min approach
Song type (adjusted vs. unadjusted)	−1.21 ± 0.95 (0.203)	**6.05 **±** 1.69** **(**<**0.001)**	−**1.45 **±** 0.45 (0.001)**	−**0.94 **±** 0.42 (0.025)**	2.77 ± 2.84 (0.34)	−0.04 ± 0.25 (0.868)
Infrastructure (pumpjack vs. control)	**2.17 **±** 0.74 (0.003)**	3.21 ± 2.31 (0.164)	−**2.04 **±** 0.87 (0.019)**	−0.84 ± 0.82 (0.31)	**8.14 **±** 3.58 (0.028)**	0.36 ± 0.50 (0.479)
Infrastructure (screw pump vs. control)	1.1 ± 0.75 (0.144)	**5.68 **±** 1.97 (0.004)**	−0.56 ± 0.71 (0.43)	−0.78 ± 0.73 (0.286)	2.23 ± 3.23 (0.493)	0.17 ± 0.48 (0.728)
CORT	−0.44 ± 0.83 (0.60)	**9.32 **±** 2.52** **(**<**0.001)**	0.70 ± 0.66 (0.292)	0.08 ± 0.68 (0.904)	0.7 ± 3.12 (0.824)	−**1.15 **±** 0.46 (0.016)**
Song type x Infrastructure (pumpjack vs. control)	0.66 ± 0.98 (0.502)	−**4.37 **±** 2.14 (0.041)**	**3.31 **±** 0.77** **(**<**0.001)**	1.3 ± 0.85 (0.126)	−**8.72 **±** 4.24 (0.05)**	−0.06 ± 0.39 (0.875)
Song type x Infrastructure (screw pump vs. control)	1.9 ± 1.01 (0.06)	−**7.02 **±** 1.78** **(**<**0.001)**	**1.41 **±** 0.52 (0.006)**	**1.37 **±** 0.58 (0.017)**	−4.64 ± 4.05 (0.264)	−0.22 ± 0.35 (0.535)
Song type x CORT	**3.66 **±** 1.34 (0.006)**	−**9.19 **±** 2.59** **(**<**0.001)**	0.93 ± 0.61 (0.13)	1.23 ± 0.68 (0.072)	−3.78 ± 3.77 (0.328)	0.12 ± 0.33 (0.73)
Infrastructure (pumpjack vs. control) x CORT	0.58 ± 0.90 (0.515)	−**7.79 **±** 2.64 (0.003)**	0.43 ± 0.85 (0.607)	−1.16 ± 0.94 (0.218)	−5.81 ± 3.83 (0.136)	0.57 ± 0.55 (0.302)
Infrastructure (screw pump vs control) x CORT	1.41 ± 1.01 (0.165)	−**9.72 **±** 2.65** **(**<**0.001)**	−0.62 ± 0.95 (0.516)	−0.3 ± 0.98 (0.756)	0.02 ± 4.34 (0.996)	**1.52 **±** 0.64 (0.023)**
Song type x Infrastructure (pumpjack vs. control) x CORT	−**4.00 **±** 1.36 (0.003)**	**9.15 **±** 2.71 (0.001)**	−1.25 ± 0.72 (0.081)	1.03 ± 0.96 (0.279)	6.71 ± 4.56 (0.155)	0.22 ± 0.41 (0.592)
Song type x Infrastructure (screw pump vs. control) x CORT	−**4.81 **±** 1.45 (0.001)**	**8.78 **±** 2.82 (0.002)**	−1.04 ± 0.7 (0.14)	−**1.93 **±** 0.88 (0.028)**	5.57 ± 5.80 (0.346)	−0.13 ± 0.53 (0.812)

Results are β ±s.e. (*P*), *P* ≤ 0.05 in bold. Categorical variables are shown with 1 vs. 0 (i.e. adjusted = 1 and unadjusted = 0), such that a positive β indicates an increase with category 1.We tested whether adjusted songs received appropriate responses in noisy environments with song played, infrastructure type and adrenocortical responsiveness as fixed effects, plus all interactions, with individual as random effect. Playbacks (*n* = 57) to individuals (*n* = 35) were divided by song and infrastructure: unadjusted songs at control sites (reference category; playbacks: *n* = 10), adjusted songs at control sites (playbacks: *n* = 9), unadjusted songs at infrastructure sites (playbacks: *n*_*pumpjacks*_* = *10; *n*_*screw pumps*_ = 9) and adjusted songs at infrastructure sites (playbacks: *n*_*pumpjacks*_ = 8; *n*_screw pumps_ = 11). Categorical variables are shown with 1 vs. 0 (i.e. adjusted = 1 and unadjusted = 0), such that a positive β indicates an increase with category 1.

**Figure 3:**
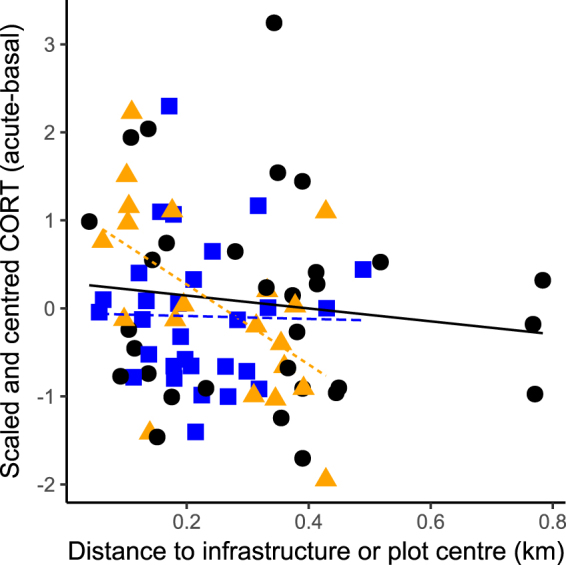
Adrenocortical responsiveness is related to distance from pumpjacks but not distance from screw pumps or distance from control site centre. Each symbol represents one male captured at control sites (black circle), pumpjacks (orange triangle) and screw pumps (blue square). Lines are predictions from linear models for control sites (black solid line), pumpjacks (orange short dashed line) and screw pumps (blue long dashed line).

Agonistic responses also varied with adrenocortical responsiveness. At control sites when song was unadjusted, increased adrenocortical responsiveness was correlated with more calls (Table 1) and reduced time to closest approach (Table 1; see Supplementary Fig. S3). Adrenocortical responsiveness was correlated with proximity to infrastructure (adult males banded in 2015–2016, *n* = 82; Fig. 3); adrenocortical responsiveness was significantly higher closer to pumpjacks (β = −3.92 ± 1.97, *P* = 0.05) but not screw pumps (β = 0.46 ± 2.08, *P* = 0.83). Further, adrenocortical responsiveness interacted with song type, such that birds with higher adrenocortical responsiveness responded more atypically to the adjusted songs at control sites (Fig. 2a; Table 1).

Although use of acoustically appropriate songs (i.e. adjusted at infrastructure sites) often restored agonistic behaviours to reference levels, the relative effectiveness of appropriate songs varied with individual physiology and infrastructure type (Table 2). Interaction slopes for adrenocortical responsiveness, infrastructure type and song type at pumpjacks were similar to those at screw pumps (Table 2). However, these similar relationships resulted in opposite consequences at the two infrastructure types. Birds with lower adrenocortical responsiveness deviated farther from reference behaviour when presented with acoustically inappropriate songs at screw pumps (number of songs and wing flicks; see Supplementary Fig. S1), whereas birds with higher adrenocortical responsiveness deviated farther from reference agonistic behaviour when presented with acoustically inappropriate songs at controls and pumpjacks (i.e. adjusted songs at control sites for number of songs [Fig. 2a; Table 1] and calls [Fig. 2b; Table 1]; to unadjusted songs at pumpjacks for number of songs [Fig. 2a] and calls [Fig. 2b]).

## Discussion

Our results demonstrate that to communicate effectively in noisy environments, Savannah Sparrow songs must be adjusted. Use of adjusted songs in control environments resulted in atypical responses, suggesting that receivers may interpret content differently between unadjusted and adjusted songs. Therefore, adjusting songs could have unintended consequences by altering perception of content^7,12,21,30^. However, adjusting songs was necessary for communicating vocalisation content and was often effective for restoring successful communication at noisy sites. The fact that birds corrected response to adjusted song in noisy conditions suggests that they were responding to adjusted features of the song, not altering response solely to a poor signal-to-noise ratio recording.

Agonistic responses to vocal cues varied, at least in part, with physiological characteristics of the individual, which in turn varied with some environmental conditions. Adrenocortical responsiveness was higher near pumpjacks than near screwpumps or at controls, suggesting that, despite their lower amplitude, pumpjacks are associated with elevated adrenocortical responsiveness, perhaps because pumpjacks induce stress^23^. Although pumpjacks are quieter than screw pumps, pumpjacks may present a stronger visual stimulus. Several studies in humans have shown that a stressor occurring in multiple sensory modalities (e.g. acoustic and visual) has a greater potential to reorient attention than a cue occurring in a single modality (i.e. acoustic)^31–33^, suggesting that pumpjacks might be disruptive because they present both acoustic and visual disruptions.

At pumpjacks, birds with high adrenocortical responsiveness benefitted most strongly from song adjustments, while birds with low adrenocortical responsiveness made fewer errors when presented with acoustically inappropriate songs; the converse was true at screw pumps. Higher overall adrenocortical responsiveness at pumpjacks was correlated with inappropriate conspecific territorial aggression behaviours to acoustically mismatched songs. The importance of vocal adjustments in the presence of noise may become increasingly important under conditions of elevated adrenocortical responsiveness (or other physiological measures correlated with it), such as those resulting from chronic environmental disturbance, which may result in other life history and resource allocation changes^34^ in addition to territorial aggression behaviours. Surprisingly, our results demonstrate that physiological characteristics that are correlated with beneficial agonistic behavioural responses in the presence of some types of anthropogenic infrastructure may be detrimental at others. Davies *et al*. (2017) suggested this as well for House Wrens^5^.

Our study suggests one reason why variation in response to anthropogenic disturbance may occur among studies, species and individuals. Adrenocortical responsiveness can be viewed as a physiological correlate of behavioural plasticity that allows individuals either to successfully cope with a given disturbance, or constrains behavioural responses resulting in detrimental effects due to chronic exposure^35,36^. Hence, differences in physiology among species^37^, with age, sex, or body condition^38–40^ and with previous exposure to disturbance^24^ may result in different responses to the same anthropogenic stimuli. Indeed, our study agrees with previous findings that different noise types can be associated with glucocorticoid responses in different ways^5^.

Nonetheless, regardless of adrenocortical responsiveness, acoustically matched songs received more appropriate agonistic behavioural responses, emphasising the importance of behavioural plasticity in anthropogenically modified environments. Intermediate levels of behavioural plasticity are considered optimal to cope with human-induced rapid environmental change^41,42^ and species or individuals that are unable to alter behaviour or respond inappropriately to novel disturbances are likely to be at a disadvantage, which may ultimately result in population declines^37^. Because different types of infrastructure have different impacts on agonistic behaviour and CORT levels^24^, land management decisions could inadvertently select for certain behavioural response patterns^43^ but these selected behaviours may not be beneficial to offspring that disperse to disparate industrial landscapes. When combined with changes in adrenocortical responsiveness due to exposure to chronic human disturbance^3^, this will result in complex selection pressures among behavioural syndromes regulated by CORT^43^ both within and among species. More study is needed to confirm causative links between physiological changes in birds in anthropogenically disturbed environments and how it affects their behaviours.

## Methods

We studied free-living adult male *Passerculus sandwichensis* (Savannah Sparrows), a grassland passerine bird. Our study (both recording of playback stimuli and playback to banded birds) was conducted in mixed grass prairies southeast of Brooks, Alberta, Canada (49° 0′ 0.004″ to 50° 53′ 56.475″ N; 110° 0′ 2.757″ W to 112° 28′ 44.473″ W).

To colour-band and take blood samples from the 35 territorial males used in the playback experiments in 2015, we lured birds to mist-nets using playbacks (www.xeno-canto.org XC153324, XC186835 and XC206187) and a decoy. Blood samples (<70 uL) were collected by brachial venipuncture in heparinised microcapillary tubes and kept iced <6 h until centrifuged and stored at −20 °C. Samples were collected in under 3 min after capture to reflect baseline circulating levels of CORT^44^ and again after a 12-min standardised stress handling protocol^45^, which reflects the ability of an individual to respond physiologically to a novel disturbance and is associated with stable behavioural traits^46^. CORT measures have been found to be repeatable within individuals in the lab^47^ and in wild populations^48,49^. The increase in CORT levels in response to handling can be more repeatable within an individual^50^, as well as having a stronger heritable component^51^, than baseline CORT. While not all studies found measures of CORT were repeatable within individuals^52^, the temporal separation in our study between capture and playback was relatively short (and within the breeding season) and therefore should be more reflective of an individual’s state at the time of playback. Plasma CORT was determined by radioimmunoassay (inter-assay variation: 14.5%, intra-assay variation: 13.4%, extraction efficiency: 113.2%) after extraction with 100% ethanol. Samples were run in duplicate tubes with a 1:6000 dilution of CORT antibody (ABIN343319; antibodies-online) and a known amount of labelled CORT (Perkin Elmer). Assay specific CORT values were determined via interpolation from a curve of serial diluted CORT standards (100–0.01 ng/mL; Steraloids) and corrected for sample volume. Adrenocortical responsiveness was not correlated with playback duration before capture (β = −0.003 ± 0.01, *P* = 0.80) or date (β = −0.009 ± 0.006, *P* = 0.14) (linear mixed model, random effect: year; *n* = 80; adult males, 2015–2016). Year was included as a random effect in the linear mixed model^53–55^ to account for potential differences in intercept between years.

We recorded the playback song stimuli as spontaneous songs May – July 2014 at control and noisy [receiving high-fidelity playback^56^ of oil well drilling noise^57^ sites using Zoom H4N Digital Recorders with built-in stereo microphones angled at 90° at maximal recording volume in uncompressed audio (WAV fles at 48 kHz sampling rate, 16-bit resolution). All recordings were made in the same region as the experiment to ensure that regional variation could not impact comparison of adjusted vs. unadjusted songs, but recordings were spatially and temporally segregated from song playback sites to ensure that receivers never heard recordings of familiar individuals. Infrastructure-free control sites, where unadjusted songs were recorded, contained only naturally occurring background noise, such as avian vocalisations and wind. Noisy sites, where adjusted songs were recorded, contained high-fidelity^56^, high-amplitude [88 dB(C) at 10 m (C-weighted time average sound pressure level for broadband sound; LC_eq_] broadcasts of oil well drilling^57^. The drilling produces frequencies that are significantly different from screw pumps and pumpjacks, but intermediate to both, in the sparrow song range (Fig. 1). The drilling noise sites had more energy in frequency bands audible to birds^58^ than ambient background noise (Fig. 1). Sparrows recorded at these drilling sites sing more loudly and at higher frequencies^57^. We chose this intermediate environment so that we could compare responses in both pumpjack and screw pump sites without favouring either treatment and expect that songs produced in an intermediate environment should be applicable to both screw pumps and pumpjacks. We created each playback stimulus with 3 songs from one individual (repeated 5 min, with natural spacing ca. 10s). We created 5 stimuli per song treatment (i.e., 5 adjusted song stimuli and 5 unadjusted song stimuli), for 10 total playback stimuli containing 30 songs from 10 individuals. Songs from both adjusted and unadjusted treatments were chosen to be typical of their category based on 5% and 95% frequency, 90% frequency bandwidth, peak frequency, aggregate entropy and average power^29,57^. Background noise was filtered below 1,500 Hz and above 12,000 Hz with a rolloff of 12 dB in Audacity^59^ and all were played at a standardised amplitude. We did not remove background noise from the adjusted songs frequencies themselves, because (1) we did not want to risk removal of song components that could not be distinguished from background noise and (2) amplitudes of background noise in song recordings were generally very low, as we used directional microphones for song recordings. This made our study more conservative, because if noise in the recordings interfered with song reception, it should result in increasingly inappropriate responses to the adjusted songs, in contrast to our prediction, that adjusting songs improves abilities of birds to communicate in noisy environments.

We played stimuli songs to resighted colour-banded males in May-July 2015, during Savannah sparrow breeding season; birds in the region were laying eggs, incubating and feeding nestlings throughout the study period. First playbacks were mean 14.0 ± 12.9s.d days (range 0–49.0; two individuals received first playback immediately before banding) from capture and CORT blood sampling, to ensure territorial response behaviour was not influenced by the stress handling protocol. When possible, each resighted male was exposed to two playbacks in randomized order: one each of adjusted and unadjusted song. However, not all males received both stimuli types, because we were unable to resight some individuals a second time. Mean 4.3 ± 6.4s.d. hours elapsed between playbacks (range 1.0–21.8) (i.e., after the bird resumed normal behaviours such as foraging and moving about its territory with no focus on the playback site). Each playback was 5 min, starting when the focal bird was heard or seen ≤50 m from the playback site. The observer estimated the focal male’s distance to the speaker in 1-m intervals and tallied agonistic behaviours in 10 s intervals. Playbacks occurred <5 h after sunrise under standardised conditions (wind <15 km/h, temp. >0 °C). Playback locations were ≤400 m of wells for noisy treatments (241 ± 103s.d. m to pumpjacks; 194 ± 79s.d. m to screw pumps; two-sided Welch’s *t* = 1.25, df = 20.6, *P* = 0.22) and ≥ 800 m from wells for control sites. These distances were chosen to correspond with quarter sections (800 × 800 m squares) that contain wells at their centre, as this is the scale at which many management decisions are made in this rural study region.

We analysed behavioural responses using generalised linear mixed-models^46–48,53^, comparing other treatments with unadjusted songs in control sites (considered the reference, or appropriate, behavioural responses). To ensure differences among treatments could not be attributed to observer, only one observer (hidden at 20 m) collected data for a given male. Required sample size was estimated *a priori* (power = 0.8); birds were sampled as logistically feasible to near that count. We fit models with Poisson distributions for behaviour counts after examining residual plots to confirm equal variance and meeting assumptions for dispersion. We met assumptions for normality and equal variance for other response variables and thus used a Gaussian distribution for those analyses. We compared sound pressure levels for noise using two-sided Satterthwaite *t*-tests for unequal and pooled *t*-tests for equal variances.

All methods were carried out in accordance with relevant guidelines and regulations under Canadian bird banding subpermits 10840A and 10840B, Canadian Wildlife Service permit #11-MB/SKL/AB-SC007 and Alberta Environment and Sustainable Research Development Research Permit #56016 and Collection Licence #56017. The experimental protocols were approved by the University of Manitoba animal care protocol F15-005.

### Data availability

The datasets generated during and/or analysed during the current study are available in the  electronic supplementary information that accompanies this paper.

## Electronic supplementary material

Supplementary information

Supplementary Datasets

Supplementary Information Code

SI_Fig.3 code
