# Supplementary material for: Noise Source and Individual Physiology Mediate Effectiveness of Bird Songs Adjusted to Anthropogenic Noise
Source: Sci Rep. 2018 Mar 2;8:3942. doi: 10.1038/s41598-018-22253-5 (PMC5834586; doi:10.1038/s41598-018-22253-5)

NOISE SOURCE AND INDIVIDUAL PHYSIOLOGY MEDIATE EFFECTIVENESS OF BIRD SONGS ADJUSTED TO ANTHROPOGENIC NOISE

Authors: Claire M. Curry, Paulson G. Des Brisay, Patricia Rosa & Nicola Koper

Supplementary Figure S1 Adrenocortical responsiveness mediates wing flicks at screw pumps but not pumpjacks. Panels marked * show significant three-way interactions relative to reference (unadjusted song at control sites). Vertical lines connect playbacks for each individual. Lines and symbols are responses to unadjusted (grey) or adjusted (black) songs. Remaining lines are model-predicted values for treatments. Reference (unadjusted songs in control sites) shown by grey dashed line.

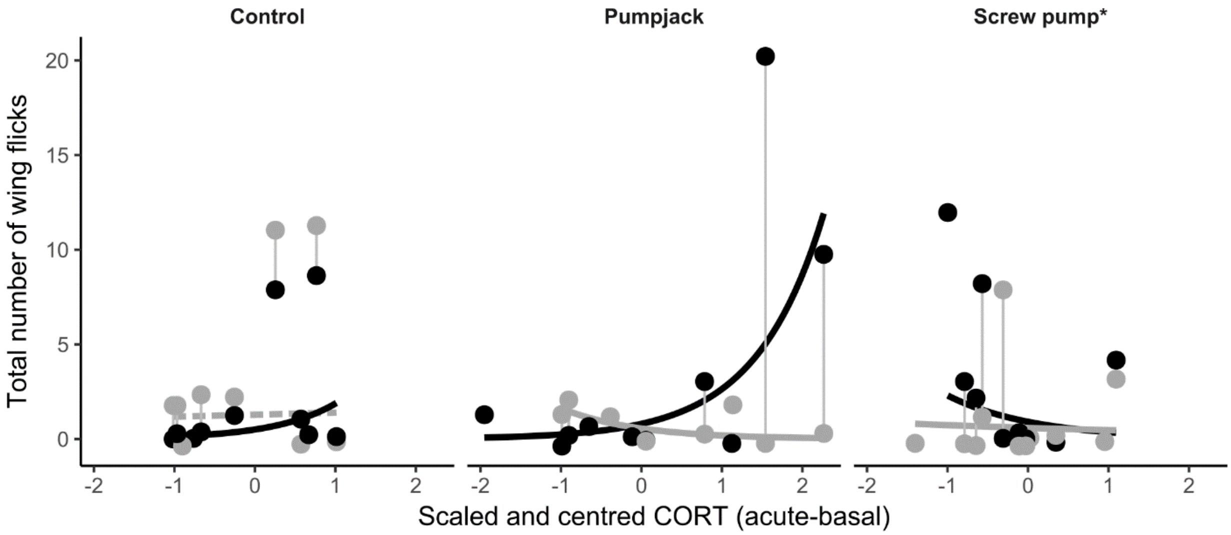

Supplementary Figure S2 Unadjusted songs are approached less closely at pumpjacks only. Panels marked * show significant two-way interactions relative to reference (unadjusted song at control sites) (Table 1). Box plots showing quartiles, median, and outliers (dots) by song type and infrastructure type for minimum approach time. Reference (unadjusted songs in control sites) shown by grey-filled box plots.

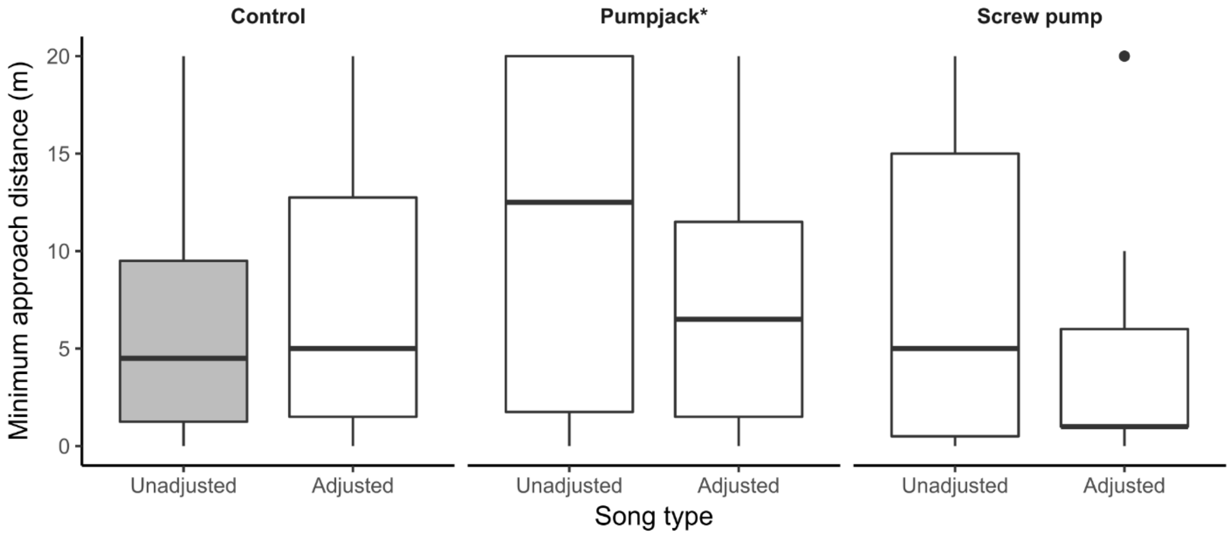

Supplementary Figure S3 Adrenocortical responsiveness reduces time to closest approach except at screw pumps. Panels marked * show significant two-way interactions between infrastructure type and CORT (Table 1). There is no significant difference between adjusted and unadjusted songs (Table 1; Table 2). Vertical lines connect playbacks for each individual. Lines and symbols are responses to unadjusted (gray) or adjusted (black) songs. Remaining lines are model-predicted values for treatments. Reference (unadjusted songs in control sites) shown by grey dashed line.

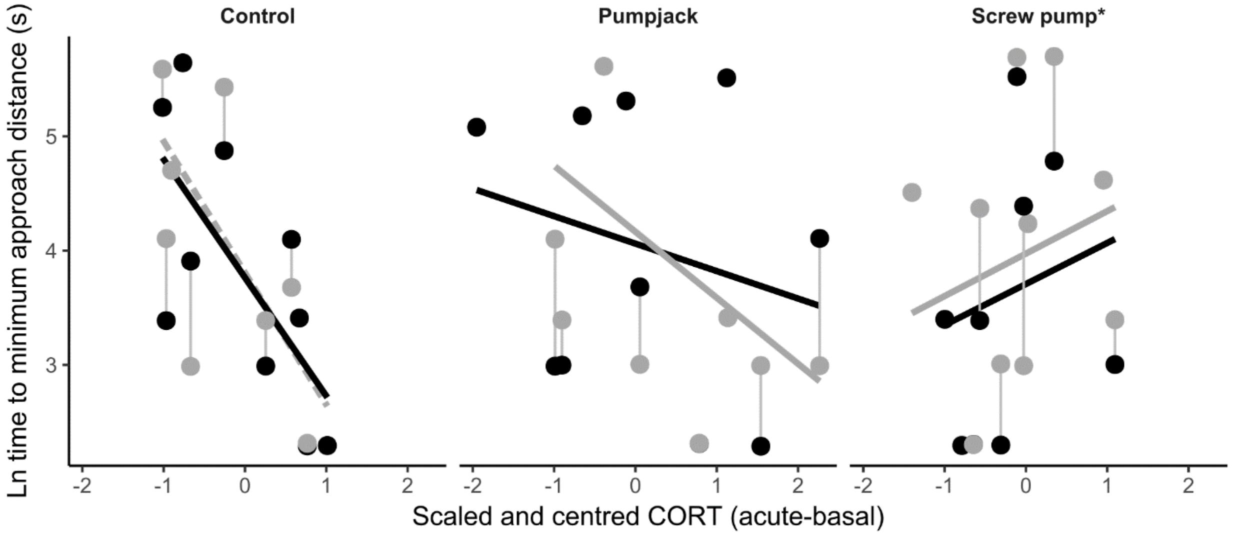

Supplement: Supplementary file 1 — Supplementary information [file 41598_2018_22253_MOESM1_ESM.pdf]
