# Supplementary material for: Noise Source and Individual Physiology Mediate Effectiveness of Bird Songs Adjusted to Anthropogenic Noise
Source: Sci Rep. 2018 Mar 2;8:3942. doi: 10.1038/s41598-018-22253-5 (PMC5834586; doi:10.1038/s41598-018-22253-5)
Supplement: Supplementary file 3 — Supplementary Information Code [file 41598_2018_22253_MOESM3_ESM.pdf]

```
#libraries
library(car)
library(checkpoint)
library(aods3)
library(dplyr)
library(ggplot2)
library(grid)
library(gridExtra)
library(lme4)
library(lmerTest)

#This file contains code for all tables and all figures except Fig. 3.

checkpoint("2017-06-07") #reproducibility

#Import data
#save excel file as .csv in working directory, then run code.
summary.SAVS.all.times.cort <- read.csv("20170719_summary_SAVS_playbacks.csv")
summary.SAVS.all.times.cort$Band.number <- as.factor(summary.SAVS.all.times.cort$Band.number)
levels(summary.SAVS.all.times.cort$Treatment) <- c("Unadjusted",
                                                    "Adjusted")
#Time between playbacks in hours
summary(summary.SAVS.all.times.cort$playback_intervals)
sd(summary.SAVS.all.times.cort$playback_intervals, na.rm=TRUE)

#Time between capture and playback in days
summary(summary.SAVS.all.times.cort$cap_playback_intervals)
sd(summary.SAVS.all.times.cort$cap_playback_intervals)

#birds under 20m
summary.SAVS.under.20m.cort<-filter(summary.SAVS.all.times.cort,
                               minApproach<999)

#distance to infrastructure mean and sd
(distance<-  dplyr::select(summary.SAVS.under.20m.cort,
                              Band.number,
                              InfrastructureType,
                              DistanceToInfrastructureKm,
                           diff.s.c) %>%
    filter(!is.na(diff.s.c),
           InfrastructureType !="CONTROL")%>%
    distinct(Band.number,
             InfrastructureType,
             .keep_all=TRUE))

t.test(DistanceToInfrastructureKm ~ InfrastructureType,
       data = distance)

  distance.summary <-   group_by(distance, InfrastructureType) %>%
    summarise(meandist=mean(DistanceToInfrastructureKm),
              sddist = sd(DistanceToInfrastructureKm))


#sample size by treatment
(sample.size<-  dplyr::select(summary.SAVS.under.20m.cort,
                              Band.number,
                              InfrastructureType,
                              Treatment,
                              diff.s.c) %>%
    filter(!is.na(diff.s.c))%>%
  distinct(Band.number,
           InfrastructureType,
           Treatment,
           .keep_all=TRUE) %>%
  group_by(InfrastructureType,
           Treatment) %>%
  summarise(samplesize=n()))

  #sample size by infrastructure only
  (sample.size.i<-  dplyr::select(summary.SAVS.under.20m.cort,
                                Band.number,
                                InfrastructureType,
                                diff.s.c) %>%
      filter(!is.na(diff.s.c))%>%
      distinct(Band.number,
               InfrastructureType,
               .keep_all=TRUE) %>%
      group_by(InfrastructureType) %>%
      summarise(samplesize=n()))

###Correlation of variables
cor(summary.SAVS.under.20m.cort[,c("totalSongs",
                                   "totalCalls",
                                   "totalAttack",
                                   "totalWingFlicks",
                                   "minApproach",
                                   "lntimeminApproach")])

###MODELS
#Tests for changes in signal CONTENT
#difference in response to control songs vs. noise songs in quiet environments (i.e. control sites only).
controlsites<-summary.SAVS.under.20m.cort[summary.SAVS.under.20m.cort$InfrastructureType=="CONTROL",]

totalSongs.content.glmer<-glmer(totalSongs~Treatment*diff.s.c+
                                    (1|Band.number),
                                data=controlsites,
                                family=poisson)

gof(totalSongs.content.glmer)
summary(totalSongs.content.glmer)

totalCalls.content.glmer<-glmer(totalCalls~Treatment*diff.s.c+
                                    (1|Band.number),
                                  data=controlsites,
                                family=poisson)
gof(totalCalls.content.glmer)
```

```
summary(totalCalls.content.glmer)

totalAttack.content.glmer<-glmer(totalAttack~Treatment*diff.s.c+
                                      (1|Band.number),
                                    data=controlsites,
                                 family=poisson)

gof(totalAttack.content.glmer)
summary(totalAttack.content.glmer)

totalWingFlicks.content.glmer<-glmer(totalWingFlicks~Treatment*diff.s.c+
                                      (1|Band.number),
                                    data=controlsites,
                                    family=poisson)

gof(totalWingFlicks.content.glmer)
summary(totalWingFlicks.content.glmer)

minApproach.content.lmer<-lmer(minApproach~Treatment*diff.s.c+
                                       (1|Band.number),
                                     data=controlsites)

plot(minApproach.content.lmer)
summary(minApproach.content.lmer)

lntimeminApproach.content.lmer<-lmer(lntimeminApproach~Treatment*diff.s.c+
                                 (1|Band.number),
                               data=controlsites)

plot(lntimeminApproach.content.lmer)
summary(lntimeminApproach.content.lmer)



#Tests for changes in signal TRANSMISSION
#difference in slope between site types and treatment
#CORT as co-variate/interaction
#bird as random effect

#Set up a priori contrast
#we compare both ppj and psp to control, so default contrasts work here.

#where models did not convert on default settings, used bobyqa optimizer and increased nAGQ to get convergence
#per help file settings for glmer and glmerControl.

totalSongs.glmer<-glmer(totalSongs~Treatment*InfrastructureType*diff.s.c+
                            (1|Band.number),
                          data=summary.SAVS.under.20m.cort,
                       family=poisson,
                       nAGQ=9,
                       control=glmerControl(optimizer="bobyqa"))

gof(totalSongs.glmer)
summary(totalSongs.glmer)

totalCalls.glmer.b<-glmer(totalCalls~Treatment*InfrastructureType*diff.s.c+
                            (1|Band.number),
                          data=summary.SAVS.under.20m.cort,
                       family=poisson,
                          nAGQ=9,
                          control=glmerControl(optimizer="bobyqa"))

gof(totalCalls.glmer.b)
summary(totalCalls.glmer.b)

totalAttack.glmer<-glmer(totalAttack~Treatment*InfrastructureType*diff.s.c+
                            (1|Band.number),
                          data=summary.SAVS.under.20m.cort,
                        family=poisson,
                        nAGQ=9,
                          control=glmerControl(optimizer="bobyqa"))

gof(totalAttack.glmer)
summary(totalAttack.glmer)


totalWingFlicks.glmer<-glmer(totalWingFlicks~Treatment*InfrastructureType*diff.s.c+
                            (1|Band.number),
                          data=summary.SAVS.under.20m.cort,
                          family=poisson,
                          nAGQ=9,
                          control=glmerControl(optimizer="bobyqa"))

gof(totalWingFlicks.glmer)
summary(totalWingFlicks.glmer)


minApproach.lmer<-lmer(minApproach~Treatment*InfrastructureType*diff.s.c+
                                 (1|Band.number),
                               data=summary.SAVS.under.20m.cort)

plot(minApproach.lmer)
summary(minApproach.lmer)

lntimeminApproach.lmer<-lmer(lntimeminApproach~Treatment*InfrastructureType*diff.s.c+
                              (1|Band.number),
                            data=summary.SAVS.under.20m.cort)

plot(lntimeminApproach.lmer)
summary(lntimeminApproach.lmer)


#SUMMARIZING RESPONSES IN TWO TABLES
#This function gets out a row for each model.
model.summary.function <- function(fm) {
  ## check that fm is an object of the "mer" class
```

```
  #stopifnot(is(fm, "merMod"))
  summaryobj<-summary(fm)
  #Bind together all the facts
  model.stats<-data.frame(gsub( " .*$", "", fm@call[2]),
                          nobs(fm),
                          ngrps(fm)[1],
                          rbind(as.vector(unlist(summaryobj$coefficients)))
  )
  #add column names
  rows<-rownames(summaryobj$coefficients)
  cols<-colnames(summaryobj$coefficients)
  parameternames<-expand.grid(rows,cols)
  parameternames$pasted<-paste(parameternames$Var1,
                               parameternames$Var2)
  all.parameters<-c("Parameter",
                    "Num obs",
                    "NumIndividuals",
                    parameternames$pasted
  )
  colnames(model.stats)<-all.parameters
  model.stats$Parameter<-gsub(".durin", "", model.stats$Parameter, fixed=TRUE)
  rownames(model.stats)<-fm@call[2]
  return(model.stats)
}


#Table 1.
models<-rbind(
  model.summary.function(totalSongs.glmer),
  model.summary.function(totalCalls.glmer.b),
  model.summary.function(totalAttack.glmer),
  model.summary.function(totalWingFlicks.glmer)
  )


table.glmer<-data.frame(
  "Nobs"=models$`Num obs`,
  "Nindividuals"=models$NumIndividuals,
  "Intercept"=paste(round(models$`(Intercept) Estimate`, digits=2),
                    "±",
                    round(models$`(Intercept) Std. Error`, digits=2),
                    " (",
                    round(models$`(Intercept) Pr(>|z|)`, digits=3),
                    ")",
                    sep=""),
  "Treatment (Adjusted vs. Unadjusted)"=paste(round(models$`TreatmentAdjusted Estimate`, digits=2),
                                        "±",
                                        round(models$`TreatmentAdjusted Std. Error`, digits=2),
                                        " (",
                                        round(models$`TreatmentAdjusted Pr(>|z|)`, digits=3),
                                        ")",
                                        sep=""),
  "Infrastructure type (PUMPJACK vs Control)"=paste(round(models$`InfrastructureTypePUMPJACK Estimate`, digits=2),
                                               "±",
                                               round(models$`InfrastructureTypePUMPJACK Std. Error`, digits=2),
                                               " (",
                                               round(models$`InfrastructureTypePUMPJACK Pr(>|z|)`, digits=3),
                                               ")",
                                               sep=""),
  "Infrastructure type (SCREWPUMP vs Control)"=paste(round(models$`InfrastructureTypeSCREWPUMP Estimate`, digits=2),
                                               "±",
                                               round(models$`InfrastructureTypeSCREWPUMP Std. Error`, digits=2),
                                               " (",
                                               round(models$`InfrastructureTypeSCREWPUMP Pr(>|z|)`, digits=3),
                                               ")",
                                               sep=""),
  "Cort"=paste(round(models$`diff.s.c Estimate`, digits=2),
                                         "±",
                                         round(models$`diff.s.c Std. Error`, digits=2),
             " (",
             round(models$`diff.s.c Pr(>|z|)`, digits=3),
             ")",
             sep=""),
  "Treatment x Infrastructure type (Adjusted vs. Unadjusted and PUMPJACK vs
Control)"=paste(round(models$`TreatmentAdjusted:InfrastructureTypePUMPJACK Estimate`, digits=2),
                                                                                 "±",
                                                                                 round(models$`TreatmentAdjusted:InfrastructureTypePUMPJACK Std.
Error`, digits=2),
                                                                                 " (",
                                                                                 round(models$`TreatmentAdjusted:InfrastructureTypePUMPJACK
Pr(>|z|)`, digits=3),
                                                                                 ")",
                                                                                 sep=""),
  "Treatment x Infrastructure type (Adjusted vs. Unadjusted and SCREWPUMP vs
Control)"=paste(round(models$`TreatmentAdjusted:InfrastructureTypeSCREWPUMP Estimate`, digits=2),
                                                                                 "±",
                                                                                 round(models$`TreatmentAdjusted:InfrastructureTypeSCREWPUMP Std.
Error`, digits=2),
                                                                                 " (",
                                                                                 round(models$`TreatmentAdjusted:InfrastructureTypeSCREWPUMP
Pr(>|z|)`, digits=3),
                                                                                 ")",
                                                                                 sep=""),
  "Treatment x Cort"=paste(round(models$`TreatmentAdjusted:diff.s.c Estimate`, digits=2),
                                                                                 "±",
                                                                                 round(models$`TreatmentAdjusted:diff.s.c Std. Error`, digits=2),
                           " (",
                           round(models$`TreatmentAdjusted:diff.s.c Pr(>|z|)`, digits=3),
                           ")",
                           sep=""),
  "Infrastructure type (PUMPJACK vs Control) x Cort"=paste(round(models$`InfrastructureTypePUMPJACK:diff.s.c Estimate`, digits=2),
                                                      "±",
                                                      round(models$`InfrastructureTypePUMPJACK:diff.s.c Std. Error`, digits=2),
                                                      " (",
                                                      round(models$`InfrastructureTypePUMPJACK:diff.s.c Pr(>|z|)`, digits=3),
                                                      ")",
```

```
                                                    sep=""),
  "Infrastructure type (SCREWPUMP vs Control) x Cort"=paste(round(models$`InfrastructureTypeSCREWPUMP:diff.s.c Estimate`, digits=2),
                                                                                 "±",
                                                                                 round(models$`InfrastructureTypeSCREWPUMP:diff.s.c Std. Error`,
digits=2),
                                                      " (",
                                                      round(models$`InfrastructureTypeSCREWPUMP:diff.s.c Pr(>|z|)`, digits=3),
                                                      ")",
                                                      sep=""),
  "Treatment (Adjusted vs Unadjusted) x Infrastructure type (PUMPJACK vs Control) x
Cort"=paste(round(models$`TreatmentAdjusted:InfrastructureTypePUMPJACK:diff.s.c Estimate`, digits=2),
                                                                                 "±",
                                                                                 round(models$`TreatmentAdjusted:InfrastructureTypePUMPJACK:diff.s.c
Std. Error`, digits=2),
                                                                                 " (",
                                                                                 round(models$`TreatmentAdjusted:InfrastructureTypePUMPJACK:diff.s.c
Pr(>|z|)`, digits=3),
                                                                                 ")",
                                                                                 sep=""),
  "Treatment (Adjusted vs Unadjusted) x Infrastructure type (SCREWPUMP vs Control) x
Cort"=paste(round(models$`TreatmentAdjusted:InfrastructureTypeSCREWPUMP:diff.s.c Estimate`, digits=2),
                                                                                                              "±",

round(models$`TreatmentAdjusted:InfrastructureTypeSCREWPUMP:diff.s.c Std. Error`, digits=2),
                                                                                       " (",

round(models$`TreatmentAdjusted:InfrastructureTypeSCREWPUMP:diff.s.c Pr(>|z|)`, digits=3),
                                                                                       ")",
                                                                                       sep="")
  )


t.table.glmer<-data.frame(t(table.glmer))
colnames(t.table.glmer)<-c("Songs",
                                      "Calls",
                                      "Attacks",
                                      "Wing Flicks")


lmermodels<-rbind(
  model.summary.function(minApproach.lmer),
  model.summary.function(lntimeminApproach.lmer))


table.lmer<-data.frame(
  "Nobs"=lmermodels$`Num obs`,
  "Nindividuals"=lmermodels$NumIndividuals,
  "Intercept"=paste(round(lmermodels$`(Intercept) Estimate`, digits=2),
                    "±",
                    round(lmermodels$`(Intercept) Std. Error`, digits=2),
                    " (",
                    round(lmermodels$`(Intercept) Pr(>|t|)`, digits=3),
                    ")",
                    sep=""),
  "Treatment (Adjusted vs. Unadjusted)"=paste(round(lmermodels$`TreatmentAdjusted Estimate`, digits=2),
                                        "±",
                                        round(lmermodels$`TreatmentAdjusted Std. Error`, digits=2),
                                        " (",
                                        round(lmermodels$`TreatmentAdjusted Pr(>|t|)`, digits=3),
                                        ")",
                                        sep=""),
  "Infrastructure type (PUMPJACK vs Control)"=paste(round(lmermodels$`InfrastructureTypePUMPJACK Estimate`, digits=2),
                                               "±",
                                               round(lmermodels$`InfrastructureTypePUMPJACK Std. Error`, digits=2),
                                               " (",
                                               round(lmermodels$`InfrastructureTypePUMPJACK Pr(>|t|)`, digits=3),
                                               ")",
                                               sep=""),
  "Infrastructure type (SCREWPUMP vs Control)"=paste(round(lmermodels$`InfrastructureTypeSCREWPUMP Estimate`, digits=2),
                                               "±",
                                               round(lmermodels$`InfrastructureTypeSCREWPUMP Std. Error`, digits=2),
                                               " (",
                                               round(lmermodels$`InfrastructureTypeSCREWPUMP Pr(>|t|)`, digits=3),
                                               ")",
                                               sep=""),
  "Cort"=paste(round(lmermodels$`diff.s.c Estimate`, digits=2),
               "±",
               round(lmermodels$`diff.s.c Std. Error`, digits=2),
               " (",
               round(lmermodels$`diff.s.c Pr(>|t|)`, digits=3),
               ")",
               sep=""),
  "Treatment x Infrastructure type (Adjusted vs. Unadjusted and PUMPJACK vs
Control)"=paste(round(lmermodels$`TreatmentAdjusted:InfrastructureTypePUMPJACK Estimate`, digits=2),
                                                                                   "±",
                                                                                   round(lmermodels$`TreatmentAdjusted:InfrastructureTypePUMPJACK
Std. Error`, digits=2),
                                                                                   " (",
                                                                                   round(lmermodels$`TreatmentAdjusted:InfrastructureTypePUMPJACK
Pr(>|t|)`, digits=3),
                                                                                   ")",
                                                                                   sep=""),
  "Treatment x Infrastructure type (Adjusted vs. Unadjusted and SCREWPUMP vs
Control)"=paste(round(lmermodels$`TreatmentAdjusted:InfrastructureTypeSCREWPUMP Estimate`, digits=2),
                                                                                   "±",
                                                                                   round(lmermodels$`TreatmentAdjusted:InfrastructureTypeSCREWPUMP
Std. Error`, digits=2),
                                                                                   " (",
                                                                                   round(lmermodels$`TreatmentAdjusted:InfrastructureTypeSCREWPUMP
Pr(>|t|)`, digits=3),
                                                                                   ")",
                                                                                   sep=""),
  "Treatment x Cort"=paste(round(lmermodels$`TreatmentAdjusted:diff.s.c Estimate`, digits=2),
                           "±",
                           round(lmermodels$`TreatmentAdjusted:diff.s.c Std. Error`, digits=2),
                           " (",
```

```
                           round(lmermodels$`TreatmentAdjusted:diff.s.c Pr(>|t|)`, digits=3),
                           ")",
                           sep=""),
  "Infrastructure type (PUMPJACK vs Control) x Cort"=paste(round(lmermodels$`InfrastructureTypePUMPJACK:diff.s.c Estimate`, digits=2),
                                                        "±",
                                                        round(lmermodels$`InfrastructureTypePUMPJACK:diff.s.c Std. Error`, digits=2),
                                                        " (",
                                                        round(lmermodels$`InfrastructureTypePUMPJACK:diff.s.c Pr(>|t|)`, digits=3),
                                                        ")",
                                                        sep=""),
  "Infrastructure type (SCREWPUMP vs Control) x Cort"=paste(round(lmermodels$`InfrastructureTypeSCREWPUMP:diff.s.c Estimate`, digits=2),
                                                        "±",
                                                        round(lmermodels$`InfrastructureTypeSCREWPUMP:diff.s.c Std. Error`, digits=2),
                                                        " (",
                                                        round(lmermodels$`InfrastructureTypeSCREWPUMP:diff.s.c Pr(>|t|)`, digits=3),
                                                        ")",
                                                        sep=""),
  "Treatment (Adjusted vs Unadjusted) x Infrastructure type (PUMPJACK vs Control) x
Cort"=paste(round(lmermodels$`TreatmentAdjusted:InfrastructureTypePUMPJACK:diff.s.c Estimate`, digits=2),
                                                                                         "±",

round(lmermodels$`TreatmentAdjusted:InfrastructureTypePUMPJACK:diff.s.c Std. Error`, digits=2),
                                                                                         " (",

round(lmermodels$`TreatmentAdjusted:InfrastructureTypePUMPJACK:diff.s.c Pr(>|t|)`, digits=3),
                                                                                         ")",
                                                                                         sep=""),
  "Treatment (Adjusted vs Unadjusted) x Infrastructure type (SCREWPUMP vs Control) x
Cort"=paste(round(lmermodels$`TreatmentAdjusted:InfrastructureTypeSCREWPUMP:diff.s.c Estimate`, digits=2),
                                                                                         "±",

round(lmermodels$`TreatmentAdjusted:InfrastructureTypeSCREWPUMP:diff.s.c Std. Error`, digits=2),
                                                                                         " (",

round(lmermodels$`TreatmentAdjusted:InfrastructureTypeSCREWPUMP:diff.s.c Pr(>|t|)`, digits=3),
                                                                                         ")",
                                                                                         sep="")
)


t.table.lmer<-data.frame(t(table.lmer))
colnames(t.table.lmer)<-c("min Approach",
                           "ln Time to Min Approach")



signal.results<-cbind(t.table.glmer,
      t.table.lmer)

#Table 1
write.csv(signal.results,
          file="signal_models.csv",
          fileEncoding="Windows-1252")


##CONTENT model summary (Extended Data Table 1)
models.content<-rbind(
  model.summary.function(totalSongs.content.glmer),
  model.summary.function(totalCalls.content.glmer),
  model.summary.function(totalAttack.content.glmer),
  model.summary.function(totalWingFlicks.content.glmer)
)


table.glmer.content<-data.frame(
  "Nobs"=models.content$`Num obs`,
  "Nindividuals"=models.content$NumIndividuals,
  "Intercept"=paste(round(models.content$`(Intercept) Estimate`, digits=2),
                    "±",
                    round(models.content$`(Intercept) Std. Error`, digits=2),
                    " (",
                    round(models.content$`(Intercept) Pr(>|z|)`, digits=3),
                    ")",
                    sep=""),
  "Treatment (Adjusted vs. Unadjusted)"=paste(round(models.content$`TreatmentAdjusted Estimate`, digits=2),
                                        "±",
                                        round(models.content$`TreatmentAdjusted Std. Error`, digits=2),
                                        " (",
                                        round(models.content$`TreatmentAdjusted Pr(>|z|)`, digits=3),
                                        ")",
                                        sep=""),
  "Cort"=paste(round(models.content$`diff.s.c Estimate`, digits=2),
               "±",
               round(models.content$`diff.s.c Std. Error`, digits=2),
               " (",
               round(models.content$`diff.s.c Pr(>|z|)`, digits=3),
               ")",
               sep=""),
"Treatment x Cort"=paste(round(models.content$`TreatmentAdjusted:diff.s.c Estimate`, digits=2),
                           "±",
                           round(models.content$`TreatmentAdjusted:diff.s.c Std. Error`, digits=2),
                           " (",
                           round(models.content$`TreatmentAdjusted:diff.s.c Pr(>|z|)`, digits=3),
                           ")",
                           sep="")
)


t.table.glmer.content<-data.frame(t(table.glmer.content))
colnames(t.table.glmer.content)<-c("Songs",
                           "Calls",
                           "Attacks",
                           "Wing Flicks")

lmermodels.content<-rbind(
  model.summary.function(minApproach.content.lmer),
```

```
  model.summary.function(lntimeminApproach.content.lmer))



table.lmer.content<-data.frame(
  "Nobs"=lmermodels.content$`Num obs`,
  "Nindividuals"=lmermodels.content$NumIndividuals,
  "Intercept"=paste(round(lmermodels.content$`(Intercept) Estimate`, digits=2),
                    "±",
                    round(lmermodels.content$`(Intercept) Std. Error`, digits=2),
                    " (",
                    round(lmermodels.content$`(Intercept) Pr(>|t|)`, digits=3),
                    ")",
                    sep=""),
  "Treatment (Adjusted vs. Unadjusted)"=paste(round(lmermodels.content$`TreatmentAdjusted Estimate`, digits=2),
                                              "±",
                                              round(lmermodels.content$`TreatmentAdjusted Std. Error`, digits=2),
                                              " (",
                                              round(lmermodels.content$`TreatmentAdjusted Pr(>|t|)`, digits=3),
                                              ")",
                                              sep=""),
  "Cort"=paste(round(lmermodels.content$`diff.s.c Estimate`, digits=2),
               "±",
               round(lmermodels.content$`diff.s.c Std. Error`, digits=2),
               " (",
               round(lmermodels.content$`diff.s.c Pr(>|t|)`, digits=3),
               ")",
               sep=""),
  "Treatment x Cort"=paste(round(lmermodels.content$`TreatmentAdjusted:diff.s.c Estimate`, digits=2),
                           "±",
                           round(lmermodels.content$`TreatmentAdjusted:diff.s.c Std. Error`, digits=2),
                           " (",
                           round(lmermodels.content$`TreatmentAdjusted:diff.s.c Pr(>|t|)`, digits=3),
                           ")",
                           sep="")
)


t.table.lmer.content<-data.frame(t(table.lmer.content))
colnames(t.table.lmer.content)<-c("min Approach",
                          "ln Time to Min Approach")



content.results<-cbind(t.table.glmer.content,
                      t.table.lmer.content)

#Extended Data Table 1.
write.csv(content.results,
          file="content_models.csv",
          fileEncoding="Windows-1252") #required to get plus or minus symbols to work right


####FIGURES
###########

#Function to get prediction lines for figure.
prediction.function<-function(behaviormerModobject,
                              infrastructuretype,
                              treatment){
  nd<-data.frame("diff.s.c"=seq(min(summary.SAVS.under.20m.cort$diff.s.c[summary.SAVS.under.20m.cort$InfrastructureType==infrastructuretype&
                                                                          summary.SAVS.under.20m.cort$Treatment==treatment],
                                              na.rm=TRUE),

max(summary.SAVS.under.20m.cort$diff.s.c[summary.SAVS.under.20m.cort$InfrastructureType==infrastructuretype&
                                                                                   summary.SAVS.under.20m.cort$Treatment==treatment],
                                              na.rm=TRUE),
                                          length.out=length(summary.SAVS.under.20m.cort$diff.s.c)),
                          "InfrastructureType"=infrastructuretype,
                          "Treatment"=treatment)
  predicted<-data.frame("y"=predict(behaviormerModobject,
                                                          newdata=nd,
                                                          type="response",
                                                          re.form=NA),#do not condition on random effects
                                              nd)
}

#Prediction lines for Figure 2.
#TotalSongs
predict.ccontrol.totalSongs<-prediction.function(totalSongs.glmer,
                                                  "CONTROL",
                                                  "Unadjusted")
predict.ppjcontrol.totalSongs<-prediction.function(totalSongs.glmer,
                                                    "PUMPJACK",
                                                    "Unadjusted")
predict.pspcontrol.totalSongs<-prediction.function(totalSongs.glmer,
                                                    "SCREWPUMP",
                                                    "Unadjusted")
predict.cnoise.totalSongs<-prediction.function(totalSongs.glmer,
                                                "CONTROL",
                                                "Adjusted")
predict.ppjnoise.totalSongs<-prediction.function(totalSongs.glmer,
                                                  "PUMPJACK",
                                                  "Adjusted")
predict.pspnoise.totalSongs<-prediction.function(totalSongs.glmer,
                                                  "SCREWPUMP",
                                                  "Adjusted")

predicts.Songs<-bind_rows(predict.ccontrol.totalSongs,
                          predict.ppjcontrol.totalSongs,
                          predict.pspcontrol.totalSongs,
                          predict.cnoise.totalSongs,
                          predict.ppjnoise.totalSongs,
                          predict.pspnoise.totalSongs)
```

```
##TOTAL CALLS

predict.ccontrol.totalCalls<-prediction.function(totalCalls.glmer.b,
                                                 "CONTROL",
                                                 "Unadjusted")
predict.ppjcontrol.totalCalls<-prediction.function(totalCalls.glmer.b,
                                                   "PUMPJACK",
                                                   "Unadjusted")
predict.pspcontrol.totalCalls<-prediction.function(totalCalls.glmer.b,
                                                   "SCREWPUMP",
                                                   "Unadjusted")
predict.cnoise.totalCalls<-prediction.function(totalCalls.glmer.b,
                                               "CONTROL",
                                               "Adjusted")
predict.ppjnoise.totalCalls<-prediction.function(totalCalls.glmer.b,
                                                 "PUMPJACK",
                                                 "Adjusted")
predict.pspnoise.totalCalls<-prediction.function(totalCalls.glmer.b,
                                                 "SCREWPUMP",
                                                 "Adjusted")

predicts.calls<-bind_rows(predict.ccontrol.totalCalls,
                          predict.ppjcontrol.totalCalls,
                          predict.pspcontrol.totalCalls,
                          predict.cnoise.totalCalls,
                          predict.ppjnoise.totalCalls,
                          predict.pspnoise.totalCalls)




##TOTAL ATTACKS

predict.ccontrol.totalAttack<-prediction.function(totalAttack.glmer,
                    "CONTROL",
                    "Unadjusted")
predict.ppjcontrol.totalAttack<-prediction.function(totalAttack.glmer,
                                                  "PUMPJACK",
                                                  "Unadjusted")
predict.pspcontrol.totalAttack<-prediction.function(totalAttack.glmer,
                                                  "SCREWPUMP",
                                                  "Unadjusted")
predict.cnoise.totalAttack<-prediction.function(totalAttack.glmer,
                                                  "CONTROL",
                                                  "Adjusted")
predict.ppjnoise.totalAttack<-prediction.function(totalAttack.glmer,
                                                    "PUMPJACK",
                                                    "Adjusted")
predict.pspnoise.totalAttack<-prediction.function(totalAttack.glmer,
                                                    "SCREWPUMP",
                                                    "Adjusted")

predicts<-bind_rows(predict.ccontrol.totalAttack,
                    predict.ppjcontrol.totalAttack,
                    predict.pspcontrol.totalAttack,
                    predict.cnoise.totalAttack,
                    predict.ppjnoise.totalAttack,
                    predict.pspnoise.totalAttack)



##TOTAL WING FLICKS
predict.ccontrol.totalWingFlicks<-prediction.function(totalWingFlicks.glmer,
                                                  "CONTROL",
                                                  "Unadjusted")
predict.ppjcontrol.totalWingFlicks<-prediction.function(totalWingFlicks.glmer,
                                                    "PUMPJACK",
                                                    "Unadjusted")
predict.pspcontrol.totalWingFlicks<-prediction.function(totalWingFlicks.glmer,
                                                    "SCREWPUMP",
                                                    "Unadjusted")
predict.cnoise.totalWingFlicks<-prediction.function(totalWingFlicks.glmer,
                                                "CONTROL",
                                                "Adjusted")
predict.ppjnoise.totalWingFlicks<-prediction.function(totalWingFlicks.glmer,
                                                  "PUMPJACK",
                                                  "Adjusted")
predict.pspnoise.totalWingFlicks<-prediction.function(totalWingFlicks.glmer,
                                                  "SCREWPUMP",
                                                  "Adjusted")

predicts.totalWingFlicks<-bind_rows(predict.ccontrol.totalWingFlicks,
                    predict.ppjcontrol.totalWingFlicks,
                    predict.pspcontrol.totalWingFlicks,
                    predict.cnoise.totalWingFlicks,
                    predict.ppjnoise.totalWingFlicks,
                    predict.pspnoise.totalWingFlicks)



##min approach distance
predict.ccontrol.minApproach<-prediction.function(minApproach.lmer,
                                                      "CONTROL",
                                                      "Unadjusted")
predict.ppjcontrol.minApproach<-prediction.function(minApproach.lmer,
                                                        "PUMPJACK",
                                                        "Unadjusted")
predict.pspcontrol.minApproach<-prediction.function(minApproach.lmer,
                                                        "SCREWPUMP",
                                                        "Unadjusted")
predict.cnoise.minApproach<-prediction.function(minApproach.lmer,
                                                    "CONTROL",
                                                    "Adjusted")
predict.ppjnoise.minApproach<-prediction.function(minApproach.lmer,
```

```
                                                          "PUMPJACK",
                                                          "Adjusted")
predict.pspnoise.minApproach<-prediction.function(minApproach.lmer,
                                                          "SCREWPUMP",
                                                          "Adjusted")

predicts.minApproach<-bind_rows(predict.ccontrol.minApproach,
                                    predict.ppjcontrol.minApproach,
                                    predict.pspcontrol.minApproach,
                                    predict.cnoise.minApproach,
                                    predict.ppjnoise.minApproach,
                                    predict.pspnoise.minApproach)

##ln time to min approach distance
predict.ccontrol.lntimeminApproach<-prediction.function(lntimeminApproach.lmer,
                                                            "CONTROL",
                                                            "Unadjusted")
predict.ppjcontrol.lntimeminApproach<-prediction.function(lntimeminApproach.lmer,
                                                              "PUMPJACK",
                                                              "Unadjusted")
predict.pspcontrol.lntimeminApproach<-prediction.function(lntimeminApproach.lmer,
                                                              "SCREWPUMP",
                                                              "Unadjusted")
predict.cnoise.lntimeminApproach<-prediction.function(lntimeminApproach.lmer,
                                                          "CONTROL",
                                                          "Adjusted")
predict.ppjnoise.lntimeminApproach<-prediction.function(lntimeminApproach.lmer,
                                                            "PUMPJACK",
                                                            "Adjusted")
predict.pspnoise.lntimeminApproach<-prediction.function(lntimeminApproach.lmer,
                                                            "SCREWPUMP",
                                                            "Adjusted")

predicts.lntimeminApproach<-bind_rows(predict.ccontrol.lntimeminApproach,
                                      predict.ppjcontrol.lntimeminApproach,
                                      predict.pspcontrol.lntimeminApproach,
                                      predict.cnoise.lntimeminApproach,
                                      predict.ppjnoise.lntimeminApproach,
                                      predict.pspnoise.lntimeminApproach)

#colors and linetypes for figures.
summary.SAVS.under.20m.cort$color <- "white"
summary.SAVS.under.20m.cort[summary.SAVS.under.20m.cort$Treatment == "Unadjusted"&
                              summary.SAVS.under.20m.cort$InfrastructureType == "CONTROL",
                            "color"] <- "gray"
summary.SAVS.under.20m.cort$Group <- "treatments"
summary.SAVS.under.20m.cort[summary.SAVS.under.20m.cort$Treatment == "Unadjusted"&
                                    summary.SAVS.under.20m.cort$InfrastructureType == "CONTROL",
                                  "Group"] <- "reference"
col <- as.character(summary.SAVS.under.20m.cort$color)
names(col) <- as.character(summary.SAVS.under.20m.cort$Group)

#color changes from: https://stackoverflow.com/questions/35279570/assign-point-color-depending-on-data-frame-column-value-r



#Figure 2
svg(file = "Figure_2.svg",
     width = 183*0.0393701,
     height = (247/3)*4*0.0393701)
#a
predicts.Songs$lines <- "solid"
predicts.Songs[predicts.Songs$Treatment == "Unadjusted"&
                 predicts.Songs$InfrastructureType == "CONTROL",
               "lines"] <- "21"
predicts.Songs$Group <- "treatments"
predicts.Songs[predicts.Songs$Treatment == "Unadjusted"&
                 predicts.Songs$InfrastructureType == "CONTROL",
               "Group"] <- "reference"
lina <- as.character(predicts.Songs$lines)
names(lina) <- as.character(predicts.Songs$Group)


predicts.Songs[predicts.Songs$Treatment == "Unadjusted", "color"] <- "darkgray"
predicts.Songs[predicts.Songs$Treatment == "Adjusted", "color"] <- "black"
predicts.Songs[predicts.Songs$Treatment == "Unadjusted", "GroupColor"] <- "Unadjusted"
predicts.Songs[predicts.Songs$Treatment == "Adjusted", "GroupColor"] <- "Adjusted"
linecol <- as.character(predicts.Songs$color)
names(linecol) <- as.character(predicts.Songs$GroupColor)

plot.a <- ggplot(data = summary.SAVS.under.20m.cort,
                   mapping = aes(x = diff.s.c,
                                 y = totalSongs,
                                 color=Treatment
                   ))+
  facet_wrap(~InfrastructureType,
             labeller = labeller(InfrastructureType = c(CONTROL = "Control",
                                                            PUMPJACK = "Pumpjack*",
                                                            SCREWPUMP = "Screw pump*")))+
  theme_classic()+
  geom_line(data=predicts.Songs,
            mapping = aes (x = diff.s.c,
                           y = y,
                           linetype = Group
            ),
            size=1.25)+
  geom_line(mapping = aes (group=Band.number),
            color="gray")+

  geom_jitter(size=3)+
  scale_color_manual(values = linecol) +
  scale_linetype_manual(values = lina) +
  theme(legend.position="none",
        strip.text.x = element_text(size=14,
                                    face="bold"),
        strip.background = element_rect(colour=c("white")),
```

```
        axis.text=element_text(size=14),
        axis.title=element_text(size=14))+
  labs(x="Scaled and centred CORT (acute-basal)",
       y="Total number of songs")

(plot.a2 <- grid.arrange(plot.a,
             top = textGrob("a",
                            x = 0,
                            y = 0.9,
                            just = c("left", "top"),
                            gp = gpar(family = "sans",
                                      font = 2))))


#b
predicts.calls$lines <- "solid"
predicts.calls[predicts.calls$Treatment == "Unadjusted"&
                 predicts.calls$InfrastructureType == "CONTROL",
               "lines"] <- "21"
predicts.calls$Group <- "treatments"
predicts.calls[predicts.calls$Treatment == "Unadjusted"&
                 predicts.calls$InfrastructureType == "CONTROL",
               "Group"] <- "reference"
linb <- as.character(predicts.calls$lines)
names(linb) <- as.character(predicts.calls$Group)


predicts.calls[predicts.calls$Treatment == "Unadjusted", "color"] <- "darkgray"
predicts.calls[predicts.calls$Treatment == "Adjusted", "color"] <- "black"
predicts.calls[predicts.calls$Treatment == "Unadjusted", "GroupColor"] <- "Unadjusted"
predicts.calls[predicts.calls$Treatment == "Adjusted", "GroupColor"] <- "Adjusted"
linecol <- as.character(predicts.calls$color)
names(linecol) <- as.character(predicts.calls$GroupColor)

plot.b <- ggplot(data = summary.SAVS.under.20m.cort,
       mapping = aes(x = diff.s.c,
                     y = totalCalls,
                     color=Treatment))+
  theme_classic()+

  facet_wrap(~InfrastructureType,
             labeller = labeller(InfrastructureType = c(CONTROL = "Control",
                                                          PUMPJACK = "Pumpjack*",
                                                          SCREWPUMP = "Screw pump*")))+
  geom_line(data=predicts.calls,
            mapping = aes (x = diff.s.c,
                           y = y,
                           linetype = Group),
            size=1.25)+
  geom_line(mapping = aes (group=Band.number),
            color="gray")+

  geom_jitter(size=3)+
  scale_color_manual(values = linecol) +
  scale_linetype_manual(values = linb) +
  theme(legend.position="none",
        strip.text.x = element_text(size=14,
                                    face="bold"),
        strip.background = element_rect(colour=c("white")),
        axis.text=element_text(size=14),
        axis.title=element_text(size=14))+
  labs(x="Scaled and centred CORT (acute-basal)",
       y="Total number of calls")
(plot.b2 <- grid.arrange(plot.b,
                         top = textGrob("b",
                                        x = 0,
                                        y = 0.9,
                                        just = c("left", "top"),
                                        gp = gpar(family = "sans",
                                                  font = 2))))
#c
summary.SAVS.under.20m.cort$color <- "white"
summary.SAVS.under.20m.cort[summary.SAVS.under.20m.cort$Treatment == "Unadjusted"&
                              summary.SAVS.under.20m.cort$InfrastructureType == "CONTROL",
                            "color"] <- "gray"
summary.SAVS.under.20m.cort$Group <- "treatments"
summary.SAVS.under.20m.cort[summary.SAVS.under.20m.cort$Treatment == "Unadjusted"&
                              summary.SAVS.under.20m.cort$InfrastructureType == "CONTROL",
                            "Group"] <- "reference"
col <- as.character(summary.SAVS.under.20m.cort$color)
names(col) <- as.character(summary.SAVS.under.20m.cort$Group)

plot.c <- ggplot(data = summary.SAVS.under.20m.cort)+
  geom_boxplot(mapping = aes(x = Treatment,
                     y = totalAttack,
                     fill = Group))+
  facet_wrap(~InfrastructureType,
             labeller = labeller(InfrastructureType = c(CONTROL = "Control",
                                                          PUMPJACK = "Pumpjack*",
                                                          SCREWPUMP = "Screw pump*")))+
  theme_classic()+
  scale_fill_manual(values = col) +
  theme(legend.position="none",
        strip.text.x = element_text(size=14,
                                    face="bold"),
        strip.background = element_rect(colour=c("white")),
        axis.text=element_text(size=14),
        axis.title=element_text(size=14))+
  labs(x="Song type",
       y="Total number of attacks")
(plot.c2 <- grid.arrange(plot.c,
                         top = textGrob("c",
                                        x = 0,
                                        y = 0.9,
                                        just = c("left", "top"),
                                        gp = gpar(family = "sans",
```

```
                                                    font = 2))))
#d

plot.d <- ggplot(data = summary.SAVS.under.20m.cort)+
  geom_boxplot( mapping = aes(x = Treatment,
                     y = totalWingFlicks,
                     fill = Group))+
  facet_wrap(~InfrastructureType,
             labeller = labeller(InfrastructureType = c(CONTROL = "Control",
                                                        PUMPJACK = "Pumpjack",
                                                        SCREWPUMP = "Screw pump*")))+
  theme_classic()+
  scale_fill_manual(values = col) +
  theme(legend.position="none",
        strip.text.x = element_text(size=14,
                                    face="bold"),
        strip.background = element_rect(colour=c("white")),
        axis.text=element_text(size=14),
        axis.title=element_text(size=14))+
  labs(x="Song type",
       y="Total number of wing flicks")

(plot.d2 <- grid.arrange(plot.d,
                         top = textGrob("d",
                                        x = 0,
                                        y = 0.9,
                                        just = c("left", "top"),
                                        gp = gpar(family = "sans",
                                                  font = 2))))

library(gtable)
library(ggplot2)
library(gridExtra)
grid.arrange(plot.a2,
             plot.b2,
             plot.c2,
             plot.d2,
             ncol = 1)
dev.off()


#Supplementary Data Fig. S1
predicts.totalWingFlicks$lines <- "solid"
predicts.totalWingFlicks[predicts.totalWingFlicks$Treatment == "Unadjusted"&
                           predicts.totalWingFlicks$InfrastructureType == "CONTROL",
               "lines"] <- "21"
predicts.totalWingFlicks$Group <- "treatments"
predicts.totalWingFlicks[predicts.totalWingFlicks$Treatment == "Unadjusted"&
                           predicts.totalWingFlicks$InfrastructureType == "CONTROL",
               "Group"] <- "reference"
lined2 <- as.character(predicts.totalWingFlicks$lines)
names(lined2) <- as.character(predicts.totalWingFlicks$Group)


predicts.totalWingFlicks[predicts.totalWingFlicks$Treatment == "Unadjusted", "color"] <- "darkgray"
predicts.totalWingFlicks[predicts.totalWingFlicks$Treatment == "Adjusted", "color"] <- "black"
predicts.totalWingFlicks[predicts.totalWingFlicks$Treatment == "Unadjusted", "GroupColor"] <- "Unadjusted"
predicts.totalWingFlicks[predicts.totalWingFlicks$Treatment == "Adjusted", "GroupColor"] <- "Adjusted"
linecol.wingFlicks <- as.character(predicts.totalWingFlicks$color)
names(linecol.wingFlicks) <- as.character(predicts.totalWingFlicks$GroupColor)

svg(file = "Figure_S1.svg",
    width = 183*0.0393701,
    height = (247/3)*0.0393701)


(plot.ed2 <- ggplot(data = summary.SAVS.under.20m.cort,
                  mapping = aes(x = diff.s.c,
                                y = totalWingFlicks,
                                color=Treatment
                  ))+
  facet_wrap(~InfrastructureType,
             labeller = labeller(InfrastructureType = c(CONTROL = "Control",
                                                        PUMPJACK = "Pumpjack",
                                                        SCREWPUMP = "Screw pump*")))+
  theme_classic()+
  geom_line(data=predicts.totalWingFlicks,
            mapping = aes (x = diff.s.c,
                           y = y,
                           linetype = Group),
            size=1.25)+
  geom_line(mapping = aes (group=Band.number),
            color="gray")+

  geom_jitter(size=3)+

    scale_color_manual(values = linecol.wingFlicks) +
    scale_linetype_manual(values = lined2) +
  theme(legend.position="none",
        strip.text.x = element_text(face="bold"),
        strip.background = element_rect(colour="white"))+
  labs(x="Scaled and centred CORT (acute-basal)",
       y="Total number of wing flicks"))
dev.off()

#Supplementary Data Fig. S2
svg(file = "Figure_S2.svg",
    width = 183*0.0393701,
    height = (247/3)*0.0393701)

(plot.ed3 <- ggplot(data = summary.SAVS.under.20m.cort)+

    geom_boxplot(data=summary.SAVS.under.20m.cort,
                 mapping = aes (x = Treatment,
                                 y = minApproach,
                                 fill = Group
```

```
                 ))+
    facet_grid(~InfrastructureType,
               labeller = labeller(InfrastructureType = c(CONTROL = "Control",
                                                          PUMPJACK = "Pumpjack*",
                                                          SCREWPUMP = "Screw pump")))+
    theme_classic()+
    scale_fill_manual(values = col) +
    theme(legend.position="none",
          strip.text.x = element_text(face="bold"),
          strip.background = element_rect(colour="white"))+
    labs(x="Song type",
         y="Minimum approach distance (m)"))
dev.off()

#Supplementary Data Fig. S3
predicts.lntimeminApproach$lines <- "solid"
predicts.lntimeminApproach[predicts.lntimeminApproach$Treatment == "Unadjusted"&
                           predicts.lntimeminApproach$InfrastructureType == "CONTROL",
                         "lines"] <- "21"
predicts.lntimeminApproach$Group <- "treatments"
predicts.lntimeminApproach[predicts.lntimeminApproach$Treatment == "Unadjusted"&
                           predicts.lntimeminApproach$InfrastructureType == "CONTROL",
                         "Group"] <- "reference"
lined4 <- as.character(predicts.lntimeminApproach$lines)
names(lined4) <- as.character(predicts.lntimeminApproach$Group)


predicts.lntimeminApproach[predicts.lntimeminApproach$Treatment == "Unadjusted", "color"] <- "darkgray"
predicts.lntimeminApproach[predicts.lntimeminApproach$Treatment == "Adjusted", "color"] <- "black"
predicts.lntimeminApproach[predicts.lntimeminApproach$Treatment == "Unadjusted", "GroupColor"] <- "Unadjusted"
predicts.lntimeminApproach[predicts.lntimeminApproach$Treatment == "Adjusted", "GroupColor"] <- "Adjusted"
linecol.lntimeminApproach <- as.character(predicts.lntimeminApproach$color)
names(linecol.lntimeminApproach) <- as.character(predicts.lntimeminApproach$GroupColor)


svg(file = "Figure_S3.svg",
    width = 183*0.0393701,
    height = (247/3)*0.0393701)

(plot.ed4 <- ggplot(data = summary.SAVS.under.20m.cort,
                 mapping = aes(x = diff.s.c,
                               y = lntimeminApproach,
                               color=Treatment))+

    facet_grid(~InfrastructureType,
               labeller = labeller(InfrastructureType = c(CONTROL = "Control",
                                                          PUMPJACK = "Pumpjack",
                                                          SCREWPUMP = "Screw pump*")))+
  theme_classic()+

  geom_line(data=predicts.lntimeminApproach,
            mapping = aes (x = diff.s.c,
                           y = y,
                           linetype = Group),
            size=1.25)+
  geom_line(mapping = aes (group=Band.number),
            color="gray")+

  geom_jitter(size=3)+

    scale_color_manual(values = linecol.lntimeminApproach) +
    scale_linetype_manual(values = lined4) +
  theme(legend.position="none",
        strip.text.x = element_text(face="bold"),
        strip.background = element_rect(colour="white"))+
  labs(x="Scaled and centred CORT (acute-basal)",
       y="Ln time to minimum approach distance (s)"))
dev.off()
```
