# Supplementary material for: Noise Source and Individual Physiology Mediate Effectiveness of Bird Songs Adjusted to Anthropogenic Noise
Source: Sci Rep. 2018 Mar 2;8:3942. doi: 10.1038/s41598-018-22253-5 (PMC5834586; doi:10.1038/s41598-018-22253-5)
Supplement: Supplementary file 4 — SI_Fig.3 code [file 41598_2018_22253_MOESM4_ESM.pdf]

```
library(checkpoint)
library(dplyr)
library(ggplot2)
library(lme4)
library(lmerTest)

#This file contains the code for Fig. 3.

checkpoint("2017-06-07") #reproducibility package

distance <- read.csv("savs_treatment_full_dataset.csv")
distance <- filter(distance,
                            recap == FALSE) #only use data from first capture/at original banding

distance <- select(distance,
                   band_num,
                   noise_dist,
                   delta_cort,
                   treatment,
                   cap_effort,
                   day,
                   year)
distance$noise_dist_km <- distance$noise_dist/1000



distance$diff.s.c<-scale(distance$delta_cort,
                     center=TRUE,
                     scale=TRUE)

distance <- na.omit(distance)

distance.cort.model.diff.alltimes<-lm(diff.s.c~treatment*noise_dist_km,
                                      data=distance)

summary(distance.cort.model.diff.alltimes)
#plot(distance.cort.model.diff.alltimes)
hist(resid(distance.cort.model.diff.alltimes))

#plot

prediction.function.distance2<-function(behaviorobject,
                                       data,
                                       infrastructuretype){
  nd<-data.frame("noise_dist_km"=seq(min(data$noise_dist_km[data$treatment==infrastructuretype],
                                                          na.rm=TRUE),
                                                      max(data$noise_dist_km[data$treatment==infrastructuretype],
                                                          na.rm=TRUE),
                                                      length.out=length(data$noise_dist_km)),
                 "treatment"=infrastructuretype)
  predicted<-data.frame("y"=predict(behaviorobject,
                                    newdata=nd,
                                    type="response"),
                        nd)
}

predict.distance.cort.c<-prediction.function.distance2(distance.cort.model.diff.alltimes,
                                                        distance,
                                                        "control")
predict.distance.cort.ppj<-prediction.function.distance2(distance.cort.model.diff.alltimes,
                                                          distance,
                                                          "pumpjack")
predict.distance.cort.psp<-prediction.function.distance2(distance.cort.model.diff.alltimes,
                                                          distance,
                                                          "screwpump")
predicts.distance.cort<-dplyr::bind_rows(predict.distance.cort.c,
                                  predict.distance.cort.ppj,
                                  predict.distance.cort.psp
)


svg(file = "Figure_3.svg",
     width = 90*0.0393701,
     height = 90*0.0393701)
ggplot(data = distance)+
  geom_jitter(mapping = aes(x = noise_dist_km,
                            y = diff.s.c,
                            shape = treatment,
                            color = treatment),
              size = 3)+
  theme_classic ()+
  geom_line (
    data=predicts.distance.cort,
    mapping = aes (y = y,
                   x = noise_dist_km,
                   lty = treatment,
                   color = treatment)
  )+
  theme(legend.position = "none")+
  scale_color_manual(values = c("black",
                                 "orange",
                                 "blue"))+
  labs(x="Distance to infrastructure or plot centre (km)",
       y="Scaled and centred CORT (acute-basal)")

dev.off()

#capture effort
delta_cort ~ cap_effort + day + (1 | year)

capeffort <- lmer(diff.s.c ~ cap_effort + day + (1|year), data = distance)
summary(capeffort)
```
